# A self-consistent approach to describe unit-cell-parameter and volume variations with pressure and temperature

**DOI:** 10.1107/S1600576721009092

**Published:** 2021-10-27

**Authors:** Ross Angel, Mattia Mazzucchelli, Javier Gonzalez-Platas, Matteo Alvaro

**Affiliations:** a IGG, CNR, Via G. Gradenigo, 6, Padova, Padova I-35131, Italy; b Mainz Institute of Multiscale Modeling and Institute of Geosciences, Johannes-Gutenberg University of Mainz, J.-J.-Becher-Weg 21, Mainz D-55128, Germany; c Departamento de Física, Instituto Universitario de Estudios Avanzados en Física Atómica, Molecular y Fotónica (IUDEA), MALTA Consolider Team, Universidad de La Laguna, La Laguna, Tenerife E-38204, Spain; d Department of Earth and Environmental Sciences, University of Pavia, Via A. Ferrata 1, Pavia I-27100, Italy

**Keywords:** equations of state, unit-cell parameters, *EosFit*, pressure

## Abstract

A method for the self-consistent description of the large variations of unit-cell parameters of crystals with pressure and temperature is presented. It employs linearized versions of equations of state (EoSs) together with constraints to ensure internal consistency. The use of polynomial functions to describe the variation of the unit-cell angles in monoclinic and triclinic crystals is compared with the method of deriving them from linearized EoSs for *d* spacings. The methods have been implemented in the *CrysFML* Fortran subroutine library. The unit-cell parameters and the compressibility and thermal expansion tensors of crystals can be calculated from the linearized EoSs in an internally consistent manner in a new utility in the *EosFit7c* program, which is available as freeware at http://www.rossangel.net.

## Abstract 2

A method is presented for the self-consistent description of the variations of unit-cell parameters of crystals with pressure and temperature.

## 1. Introduction

The measurement of the response of the unit-cell parameters and therefore the volume of crystals to hydro­static pressure (*P*) and temperature (*T*) provides fundamental information about the nature and anisotropy of the bonding within the structures of crystals. The variation of the unit-cell volume, molar volume and density of a material with pressure and temperature is described by its equation of state (EoS). The formulae that relate the volume or density of a material to the applied pressure are based on various assumptions. They include assumed interatomic potentials and structural geometries, or an assumed relationship between parameters and pressure (*e.g.* the Murnaghan and Tait EoSs), or an assumed relationship between the strain arising from compression and the free energy of the solid (*e.g.* the Birch–Murnaghan EoS). Thermal EoSs include various purely parametric forms and those such as the Mie–Grünesien–Debye EoS that involve assumptions about the phonon density of states of the solid and its contribution to the heat capacity and thus the thermal pressure. A full review of EoSs and these issues has been given by Anderson (1995 ▸).

An EoS for volume (and therefore density) describes isotropic properties. Such EoSs do not describe the anisotropy of the response of crystal structures to pressure or temperature, which is described by the second-rank tensors of thermal expansivity and compressibility. While the variations of individual elastic moduli of a crystal (a fourth-rank tensor) with *P* and *T* are related to the Helmholtz free energy (*e.g.* Stixrude & Lithgow-Bertelloni, 2005 ▸), the expression involves derivatives of the individual elastic moduli that are often not available, while the equations cannot easily be reduced to describe the anisotropic thermal expansion and compressibility under hydro­static pressure.

Therefore, the variations of individual unit-cell parameters of crystals as *P* and *T* are changed have been described by using linearized equations of state in which the individual cell parameters are cubed and then fitted using the volume EoS (*e.g.* Angel, 2000 ▸). When applied to unit-cell parameters, these have been called ‘axial EoS’. Alternatively, the equations for thermal expansion or compressibility can be converted to equivalent linear forms to fit unit-cell data (*e.g.* Kroll *et al.*, 2014 ▸; Murshed *et al.*, 2015 ▸). These methods can be extended to any direction within the unit cell. In most cases they provide an accurate description of the variation of the individual unit-cell parameters with* P *and/or *T*, and yield linear compressibilities and moduli that agree with those determined by direct measurements of the elastic tensors. The advantage of these approaches is that the underlying theory of volume EoSs is well developed and their limitations in parameter and *P* and *T* space are well understood (*e.g.* Holzapfel, 2001 ▸; Anderson, 1995 ▸; Angel *et al.*, 2019 ▸), so linearized EoSs can be extrapolated beyond the range of data with more confidence and justification than simple polynomial functions of the unit-cell parameters with *P* and *T*.

However, the volume and the unit-cell parameters of a crystal are not independent of one another, so the use of independent equations for the variation of the unit-cell parameters and the volume introduces additional false degrees of freedom into the description of the behaviour of the crystal. Furthermore, the relationship between the volume and the unit-cell parameters imposes specific constraints that relate the bulk modulus, 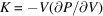 , and its derivatives to the axial linear moduli, 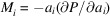 , and their derivatives. As we prove below, except for cubic crystals, as pressure is increased the different axial moduli increase at different rates, and the symmetry constraints relating the bulk modulus and its derivatives to the linear moduli and their derivatives are violated if conventional volume and axial EoSs are used to describe the variation of the moduli with pressure. The same problem occurs with the thermal expansion coefficients of the volume and the unit-cell parameters. Therefore, describing the anisotropic changes with *P* and *T* of a unit cell and its volume with independent EoSs is not physically consistent.

There is an additional but distinct difficulty that arises when the crystal has monoclinic or triclinic symmetry. The use of an axial EoS does not address how to describe the variation of unit-cell angles with *P* and *T*, or composition. This can be achieved by using the strain tensor and its derivative tensors of compressibility and thermal expansivity (Nye, 1957 ▸; Ohashi & Burnham, 1973 ▸). These define the instantaneous variation of all of the unit-cell parameters of a crystal, including the unit-cell angles. The linearized EoS defines the components of these property tensors that correspond to the axes of the unit cell, but there is no independent underlying theory that defines how the other tensor components vary with *P* and *T* in monoclinic and triclinic crystals. This means that a strain tensor analysis cannot be used to extrapolate the behaviour of the crystal to *P* and *T* ranges beyond that of the data. Furthermore, when two unit-cell determinations are made at both different *P* and different *T*, the strain is defined but the partitioning of the strain into *T*- and *P*-induced components is not, so the data cannot be interpreted in terms of compression and thermal expansion. And when the unit-cell angles of monoclinic and triclinic crystals change significantly from one measurement to another, the strain tensors do not provide a unique description of the unit-cell-parameter variation or of the directions of the principal axes of the strain, which include the directions of greatest and least strain (*e.g.* Paufler & Weber, 1999 ▸; Knight, 2010 ▸; Langreiter & Kahlenberg, 2015 ▸). This is important when trying to relate the directions of greatest or least compressibility or thermal expansion to the crystal architecture and thus the bonding within the crystal structure.

All of these issues have recently become more important in mineralogy as host-inclusion piezobarometry has been developed. This technique uses the stress and the strain measured in an inclusion crystal trapped inside a host mineral to determine the *P* and *T* under which the inclusion was trapped. In its simplest isotropic approximation only the volume equations of state of the minerals are required (Angel, Mazzucchelli *et al.*, 2014 ▸). More recent developments of the method for anisotropic phases (Alvaro *et al.*, 2020 ▸; Mazzucchelli *et al.*, 2019 ▸; Gonzalez *et al.*, 2021 ▸) require an accurate, precise and internally consistent description of how the unit-cell parameters of both the host and inclusion phases change with pressure and temperature. If the equations for the unit-cell parameters of a phase do not result in exactly the same volume as that calculated from its volume EoS, then the small discrepancies can propagate into significant errors in the calculation of entrapment conditions.

In this paper we present a simple phenomenological approach to describe consistently the variation of the unit-cell parameters of crystals with *P* and *T*. It is based on using axial EoSs to describe the cell-parameter variations, but with constraints to ensure that full internal consistency is maintained between the predicted unit-cell parameters and volume. We then use the method of Paufler & Weber (1999 ▸) to calculate the compressibility and thermal expansivity tensors directly from these linearized EoSs at any *P* and *T*. This allows the elastic behaviour of any direction in the crystal structure to be described in a consistent manner and the principal axes of the tensors, which include the directions of greatest and least strain, to be unambiguously defined. We also document how this method is implemented in a new version of the *eos* module in the *CrysFML* software library and within the established *EosFit7c* program for EoS calculations (Angel, Alvaro & Gonzalez-Platas, 2014 ▸). A new utility in the *EosFit7c* program allows the user to perform all of the data analysis and EoS calculations described in this paper. The program is freely available in compiled form for Windows, Linux and macOS operating systems at http://www.rossangel.net, together with example data sets and complete documentation. The *CrysFML* subroutine library (Rodriguez-Carvajal & Gonzalez-Platas, 2003 ▸) is open source and is available at https://code.ill.fr/scientific-software/crysfml. The architecture of the *EosFit7c* program also allows it to be called from other software such as MATLAB (The MathWorks Inc., Natick, MA, USA) to perform EoS calculations without the need to cross-compile software directly with the *CrysFML* library.

## 2. Constrained equations of state

### 2.1. The theoretical basis

The fundamental constraint on the unit-cell parameters of a crystal is that they must obey the relationship

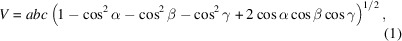

which for ease of notation we will write as

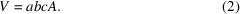

The *A* in (2) represents the entire square-root expression in (1), which involves only the unit-cell angles. The derivative of (2) relates changes in the volume to the changes induced in the unit-cell parameters:

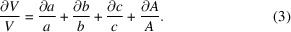




As an example, it follows that the volume compressibility 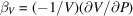 is also related to the compressibilities of the individual cell parameters [*e.g.* 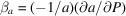 ] as

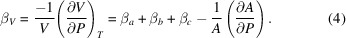




An equivalent expression exists for thermal expansivity, 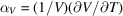 . In crystal systems of orthorhombic and higher symmetry, the unit-cell angles are fixed by symmetry and the volume compressibility is just the sum of the linear compressibilities of the unit-cell axes:

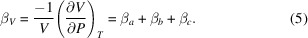




Further differentiation of (4) gives the relationship between the first pressure derivatives of the compressibilities of crystals:

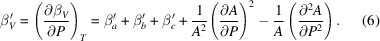




The volume EoS is usually parameterized and expressed in terms of the isothermal Reuss bulk modulus, 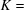 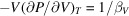 . The linear compressibilities are the inverse of the corresponding isothermal Reuss linear moduli, *e.g.* 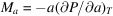 , so from (4) the relationship between these moduli must always be

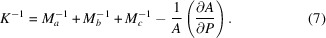




The relationship between the pressure derivative of any individual compressibility and the pressure derivative of its corresponding modulus 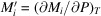 is

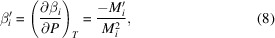

so that the relationship (6) between the first pressure derivatives of the compressibilities can be expressed in terms of moduli and their pressure derivatives as

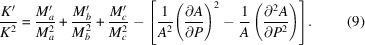




Thus, in order to be completely consistent in the description of the properties of a unit cell, the linearized EoSs of the unit-cell axes and the volume EoS must obey not only the relationship given by (1) but also those expressed in equations (7) and (9), at all pressures.

Examination of these equations shows that this is only possible for three special cases: first, when the compressibilities, moduli and their derivatives are identical for all directions in the crystal, which is only true in cubic crystals; second, when the pressure derivatives of the moduli are all zero, so that all of the moduli remain equal to their room-pressure values at all pressures; and third, when the pressure derivatives ( 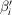 ) of the compressibilities are independent of pressure, which allows the constraints expressed in equations (4) and (6) to be met at all pressures. However, the last two cases are physically unrealistic, as non-zero pressure derivatives describe the stiffening of the structure under compression, and the rate of this stiffening changes with pressure, which is an intrinsic property of all commonly used EoSs.

**Figure 1:**
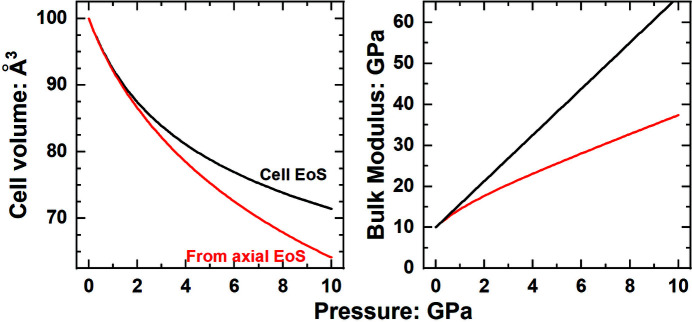
Variation of the unit-cell volume and bulk modulus of a tetragonal crystal calculated with the EoS parameters listed in Table 1 ▸. The EoS parameters obey the symmetry constraints between volume and axial properties at *P* = 0, but under compression the volume and bulk modulus calculated from the axial EoS deviate significantly from those from the volume EoS. Murnaghan EoS used for both axes and volume.

As a simple example, the Murnaghan EoS assumes a linear variation of *K* (or *M* for the cell axes) with pressure, corresponding to constant values of *K*′ and *M*′. However, equation (8) shows that constant *K*′ and *M*′ do not correspond to constant values of the pressure derivatives of the compressibilities 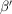 . Therefore, the Murnaghan EoS can only meet the criterion set by (4) at a single pressure. Even if all of the axes have the same value of *M*′, equation (9) shows that *K*′ for the volume calculated from the unit-cell-parameter EoS with constant *M*′ will vary with pressure, because the values of *M* increase with increasing pressure. But the corresponding Murnaghan EoS for the volume will have, by definition, constant *K*′. The consequences of this are shown in Fig. 1 ▸, with a simple example of the volume variation of a soft tetragonal crystal predicted from the axial EoSs for the unit-cell parameters and the EoS for the volume. The parameters *V*_0_, 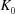 and 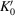 of the volume EoS at the reference conditions (Table 1 ▸) are exactly those required by the constraints given in equations (1)–(9) above. However, although all of the elastic properties of the cell and volume obey these constraints at the reference conditions, they do not obey them at any other pressure. For example, the axial moduli at 1 GPa will be 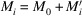 , so 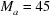 GPa and 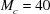 GPa, from which one obtains [equation (7)] a bulk modulus of *K* = 14.4 GPa, whereas the bulk modulus predicted by the Murnaghan EoS for the volume (Table 1 ▸) would be 15.625 GPa. The Birch–Murnaghan third-order EoS produces similar discrepancies in the bulk modulus values. Using the same room-pressure parameters (Table 1 ▸), it yields for a pressure of 1 GPa values of 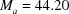 GPa and 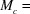 38.09 GPa, which imply a bulk modulus of *K* = 13.99 GPa, whereas that from the volume EoS is 15.32 GPa. Therefore, the volume and bulk modulus predicted from the volume EoS show increasing divergence from the values predicted by the axial EoS as pressure is increased (Fig. 1 ▸); the same behaviour is exhibited by all other EoS formulations.

**Figure 2:**
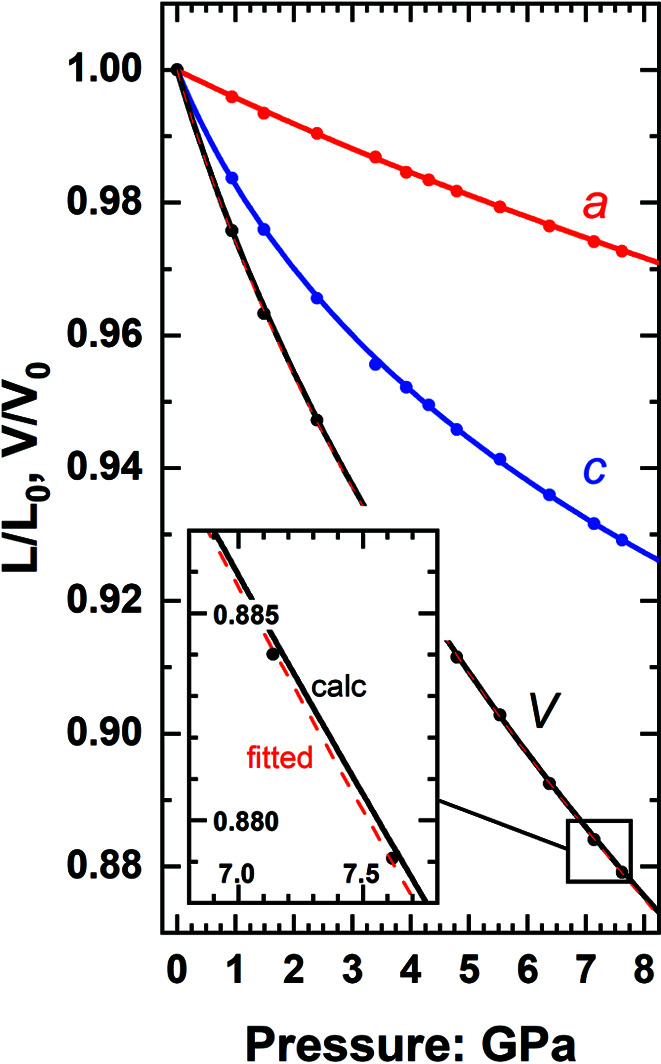
Evolution with pressure of the unit-cell parameters and volume of trigonal TiS_2_. Data are from Allan *et al.* (1998 ▸). The lines for *a* and *c* are the axial Birch–Murnaghan EoSs fitted to the data, and the black solid line is the volume variation calculated from these two EoSs. It is almost indistinguishable from the EoS fitted directly to the *P*–*V* data (red dashed line).

The rate at which these differences in volumes and bulk moduli increase with pressure depends on the bulk modulus, the elastic anisotropy of the crystal [equations (4) and (7)] and in particular on the anisotropy of the pressure derivatives of the axial compressibilities [equations (6) and (9)]. Therefore, the differences in volume calculations depend on the amount of compression (fractional volume decrease) applied to the crystal, not on the pressure itself. The difference in predicted volumes for the example in Fig. 1 ▸ reaches 1% at approximately 15% volume compression, which occurs at a pressure of about 2 GPa for this example, corresponding to ∼*K*_0_/5. Thus, a stiffer material with the same degree of anisotropy will only show similar differences in volumes at higher pressures. For example, the volume differences for TiS_2_ are only slightly larger than the estimated experimental uncertainties up to pressures of 9 GPa (Fig. 2), because it has a 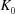 three times larger than the example in Fig. 1 ▸. Therefore, whether or not the effects of anisotropy on the calculation of cell parameters and volumes is significant depends not only on the elastic properties of the crystal but also on the precision of the experimental data, the pressure and compression range being considered, and the precision and internal consistency required in calculations.

Clearly, the unit-cell volume, the unit-cell parameters and their derivatives [equations (1)–(9)] are not independent quantities, and therefore the description of their behaviour with *P* and/or *T* by independent EoSs introduces false additional degrees of freedom. We have also demonstrated here that, if the variations of all of the unit-cell parameters and volume are described by realistic EoSs, then the constraints on the relationship between the elastic properties of the volume and the unit-cell parameters cannot be met exactly at all pressures except in special cases. Therefore, it is not physically consistent to describe the anisotropic evolution of a unit cell with *P* and *T* with completely independent EoSs. The following examples, grouped by crystal system, show how this problem can be overcome and a fully self-consistent description of the variation of the parameters of a unit cell of a crystal can be obtained. Most of the examples illustrate the problem in the context of isothermal compression, for which the effects are largest. However, exactly the same methodology can be applied to thermal expansion or, in general, an EoS describing how the unit-cell parameters change with *P* and *T*, as illustrated below with the example of quartz.

### 2.2. Cubic crystal system

For cubic crystals the general relationship (1) between the unit-cell edge *a* and the volume is simplified to 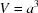 , and it follows from the constraint equations given above that the elastic properties of all of the unit-cell edges have the same relationship to those of the volume. Thus,

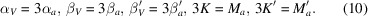

This means that the variation of the unit cell of a cubic crystal can be described equally well with either a linearized EoS for the unit-cell parameter *a* or an EoS for the volume with the parameter relations given in (10), which will then hold for all temperatures and pressures, unlike the example shown in Fig. 1 ▸. In addition, because the thermal expansion and compressibility are second-rank tensor properties, these are identical for all directions in a cubic crystal.

### 2.3. Uniaxial crystal systems

In these crystal systems the values of the unit-cell angles are fixed by the symmetry so the angle factor *A* [equations (1) and (2)] is 1.0 in the tetragonal system and *A* = sin(120°) for the trigonal and hexagonal systems in their conventional settings. Being constant, the angle factor *A* does not contribute to the volume derivatives as defined by equation (3). Therefore, for the uniaxial crystal systems the variation in the unit-cell parameters can be described by fitting EoSs to any two of *a*, *c* and *V*, setting the EoS of *b* to be equal to that of the *a* axis, and calculating the properties of the third symmetrically independent parameter from the other two. Fig. 2 ▸ shows the unit-cell variation of TiS_2_ with pressure (Allan *et al.*, 1998 ▸) treated in this way; this is used as an example because TiS_2_ has a layered structure with very strong anisotropy in both the axial moduli and their pressure derivatives, and it therefore provides a challenging test. The unit-cell volume calculated from the axial Birch–Murnaghan EoSs for *a* and *c* is indistinguishable on the scale of the figure from the volume EoS fitted directly to the volume data. In detail, the average misfit to the data quantified as (*P*_obs_ − *P*_calc_) is 0.024 GPa from the unit-cell EoS and 0.015 GPa from the direct volume EoS. From room pressure to 5 GPa, in the middle of the data set where the elastic parameters are best constrained, the difference in *K* and *K*′ between the two descriptions of the volume is less than 1.5 e.s.d., but then increases to the level of 2 e.s.d. at 8 GPa. Thus, for this very anisotropic example, the fit of the axial EoS represents the volume properties almost as well as the direct fit to the volume data.

**Figure 3:**
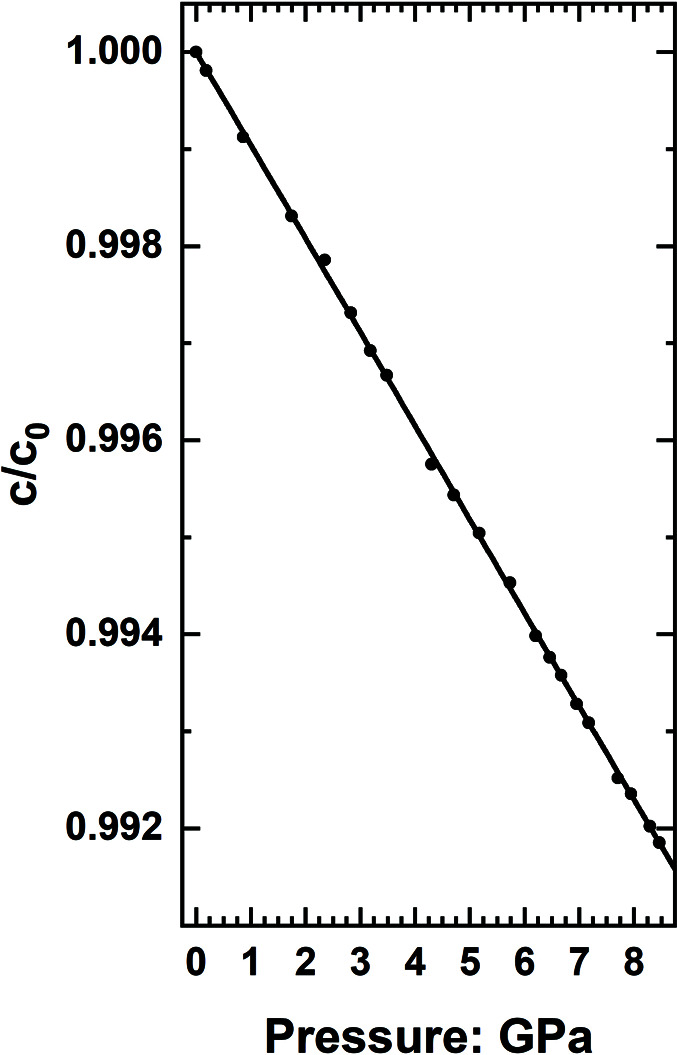
Pressure evolution of the unit-cell parameter *c* of zircon. The symbols are the measured data, and the line is the predicted evolution calculated from the EoSs fitted to the *a*-cell parameter and the volume. Data and EoS from Ehlers *et al.* (2022 ▸).

The mineral zircon, ZrSiO_4_, has tetragonal symmetry and is also strongly elastically anisotropic with the *c* axis being almost twice as stiff as the *a* and *b* axes (*e.g.* Özkan *et al.*, 1974 ▸; Ehlers *et al.*, 2022 ▸). This makes it challenging to determine the linear modulus of the *c* axis from just measurements of the unit-cell parameters under pressure. Fig. 3 ▸ shows that the behaviour of the *c* axis of zircon can be well represented by calculating it as *c* = *V*/*a* ^2^, with the values of *V* and *a* obtained from their corresponding EoSs fitted to the experimental data (Ehlers *et al.*, 2022 ▸). The calculated *c*-axis variation gives a value of *M*_*c*0_ that is within 1 e.s.d. of the value determined by a combined fit to elasticity and compressional data (Ehlers *et al.*, 2022 ▸), and also reproduces the slight softening ( 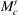 < 0) from 0 to ∼4 GPa that is apparent in the data (Fig. 3 ▸).

**Figure 4:**
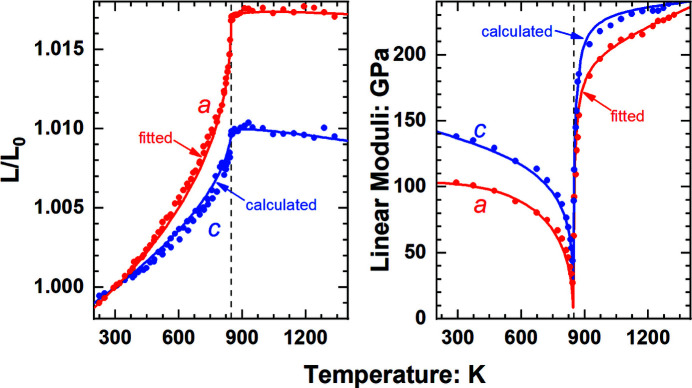
Variation of the unit-cell parameters and linear moduli of quartz with temperature at room pressure. Data points are from Carpenter, Salje, Graeme-Barber, Wruck *et al.* (1998 ▸) and Lakshtanov *et al.* (2007 ▸). Red lines are a linear EoS for the *a* axis fitted to the same *PT* data as used to determine the volume EoS (Angel *et al.*, 2017 ▸). The blue lines for the *c* axis are not fitted to the data, but are calculated from the volume and *a*-axis EoSs. The α–β phase transition in quartz is marked by the vertical dashed line.

A further stringent test of the method is provided by the extreme values of properties in the neighbourhood of continuous displacive phase transitions (*e.g.* Carpenter, Salje & Graeme-Barber, 1998 ▸; Carpenter & Salje, 1998 ▸). In quartz, the α–β phase transition at ∼848 K and room pressure (*e.g.* Carpenter, Salje, Graeme-Barber, Wruck *et al.*, 1998 ▸) is accompanied by almost infinite thermal expansion and compressibilities in the low-temperature α phase, the latter corresponding to the softening of the isothermal bulk modulus and linear moduli to zero. Fig. 4 ▸ shows that the behaviour of the *c* axis calculated as *c* = *V*/*a* ^2^sin(120°) from the volume and *a*-axis EoSs is reproduced just as well as the *a*-axis properties are predicted by the EoS fitted directly to the *a*-axis data. In particular, this calculation for the *c* axis captures the reduction of the linear modulus *M_c_* as the transition is approached in the α phase, the rapid recovery of this modulus in the β phase, and the negative thermal expansion of the *c* axis in the β phase.

### 2.4. Orthorhombic crystal system

**Figure 5:**
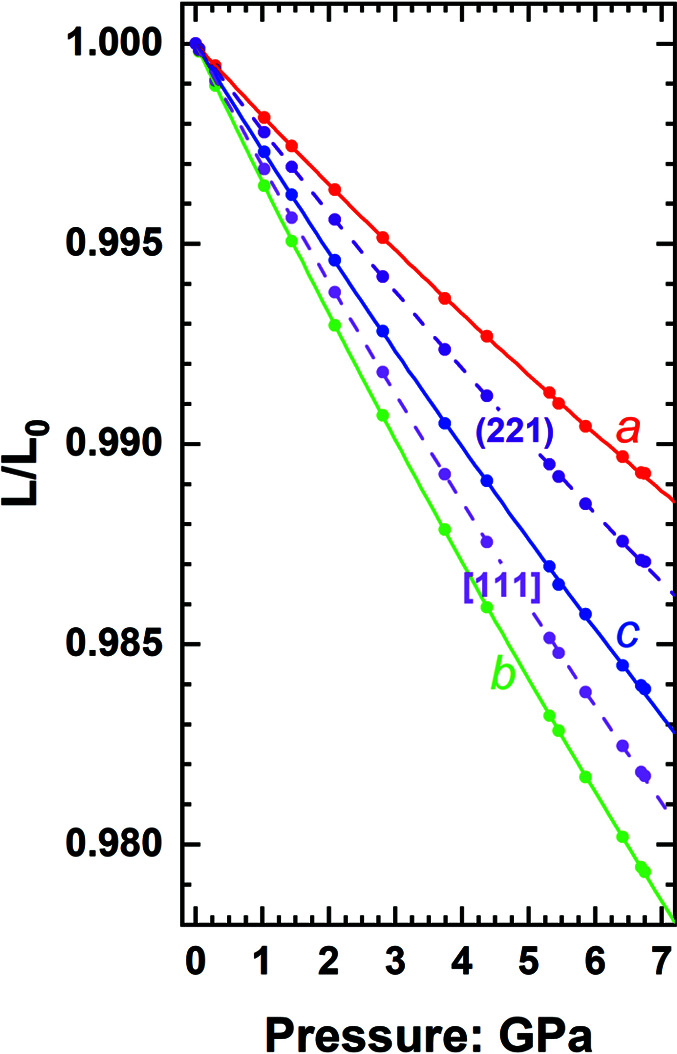
Pressure evolution of the unit-cell parameters of orthorhombic forsterite, Mg_2_SiO_4_. Data points for cell parameters are from Poe *et al.* (2010 ▸), and the corresponding lines are the fits of linearized EoSs to these data. Data points for [111] and (221) were calculated directly from the measured cell parameters at each pressure, and the corresponding dashed lines are calculated from the axial EoS.

For orthorhombic crystal systems the unit-cell angles are constrained to be 90°, the factor *A* = 1.0, and the volume of the unit-cell is simply the product *V* = *abc*. Therefore, the anisotropy of the elastic properties of the unit cell of an ortho­rhombic crystal can be described completely by specifying the EoSs of three of these quantities and calculating the fourth from them. As for crystal systems of higher symmetry, this also allows the compressibility of any direction in the crystal to be calculated at any *P* and *T* from the EoS. As an example, Fig. 5 ▸ shows the pressure variation of the length of the [111] lattice vector and the *d* spacing of the (221) planes in pure forsterite, Mg_2_SiO_4_, calculated using the axial EoSs for the unit-cell axes.

### 2.5. Monoclinic crystal system

**Figure 6:**
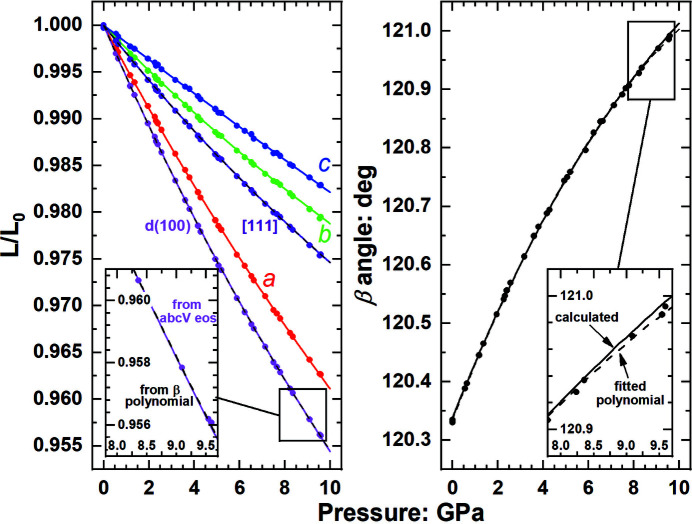
Unit-cell-parameter and β-angle variation of monoclinic SiO_2_, coesite, with pressure (Angel *et al.*, 2001 ▸). Solid lines for *a*, *b* and *c* are the fitted linearized EoSs. Together with the EoS fitted to the unit-cell volume, these allow the pressure evolution of both the unit-cell angle and directions in the unit cell (data points) to be accurately reproduced (solid lines). This includes *d*(100) which is a principal axis of compression of the structure and softer than any individual cell edge. The dashed line for the β angle is a second-order polynomial fit to the data. The dashed lines for *d*(100) and [111] calculated from this polynomial and the axial EoS are indistinguishable from values calculated from the axial EoS plus the volume EoS.

We use the conventional setting of the monoclinic crystal system with the *b* axis being unique to describe our methodology for monoclinic crystals. Equivalent expressions for the relationships between cell parameters follow if a different cell setting with a different unique axis is chosen. For *b* unique, the angle factor in equations (1) and (2) is 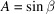 , and the unit-cell parameters and volume are related by 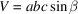 . It follows that the unit cell can be completely described by four of these five parameters. Self-consistent calculations can therefore be achieved in different ways. We find that for precise data with β angles greater than 100° both the angle and the lengths of all directions can be well reproduced (Fig. 6 ▸) by using EoSs fitted to the volume and the cell parameters, and calculating 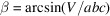 . Note that because of the trigonometric identity 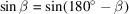 one has to specify whether the β angle is obtuse or acute (greater or less than 90°).

**Figure 7:**
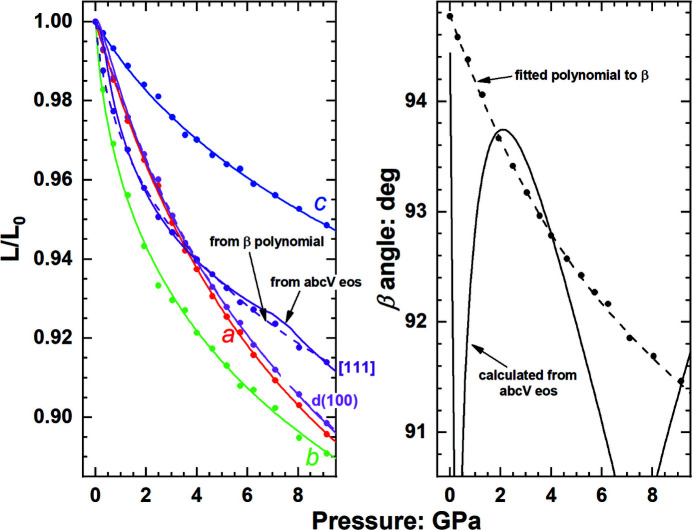
Unit-cell-parameter and β-angle variation of monoclinic C_4_H_4_N_3_ClCuI with pressure (unpublished data). Solid lines for *a*, *b* and *c* are the fitted axial EoSs. The β angle calculated from these and the volume EoS is shown as a solid line and does not match the measured data because of the uncertainties in the EoS parameters of the axial EoSs. These cause the lengths of vectors not in the (010) plane, such as [111], to have predicted variations with pressure (solid line) that are unrealistic. The use of a polynomial function to describe the evolution of the β angle with pressure (dashed line) together with the same axial EoS reproduces (dashed lines) the measured variation of *d*(100) and [111] with pressure.

The uncertainty in the β angle (in degrees) calculated in this way is 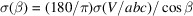 , where 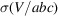 is the combined uncertainty in 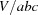 . The factor of 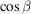 in the denominator ensures that the uncertainty in β angle approaches infinity as β approaches 90°; the value calculated from the EoS becomes less reliable and more sensitive to uncertainties in 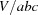 to the extent that the value of β can be completely dominated by fitting errors and noise in the EoS and the underlying data, together with numerical precision in the computer code. The predicted behaviour of both the β angle and consequently some directions in the crystal can become completely wrong and unphysical as shown in Fig. 7 ▸. An additional problem is that uncertainties in the EoS parameters can sometimes result in the ratio 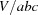 calculated from their EoS being greater than 1, for which no value of β can be defined.

This is especially a problem in soft materials such as molecular crystals, in which the rapid increase in moduli with pressure and strong anisotropy in *M*′ means that the volume variation with pressure includes a significant component from the variation in the β angle [equation (3)], which in turn means that the EoSs do not fit the data as well as for stiffer materials over the same pressure range (compare Figs. 6 ▸ and 7 ▸). Because 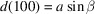 , one can also describe the unit cell by an EoS for this *d* spacing, or an EoS for 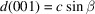 , plus EoSs for three of *a*, *b*, *c* and *V*. However, when the β angle approaches 90°, the evolution of the *d* spacings with *P* and *T* approaches that of the corresponding unit-cell axes *a* and *c*. The value of the β angle obtained from the two EoSs as 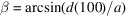 then suffers the same unreliability just described.

Because the problem is in the recovery of the β angle from the calculated cell parameters, an alternative approach is to describe the variation of the angle directly in terms of *P* and *T*. There is no constraint from theories of elasticity or EoSs that define how unit-cell angles should change with *T* or *P*, so a phenomenological approach can be adopted. For example, a polynomial of the angle in *P* and *T* or a polynomial of a trigonometric function or functions of the angle can be used. The only requirement is that the function chosen provides an invertible relationship that defines a unique angle for a given *P* (or *T*) and a unique *P* for a given angle and *T*. Then, given that the β angle is defined, EoSs for three of the four parameters *a*, *b*, *c* and *V* are required to complete the description of the anisotropic properties of the unit cell. While this loses the advantages of using only the EoS, especially with respect to extrapolation to conditions beyond the range of the data, Figs. 6 ▸ and 7 ▸ show that, if the variation of the monoclinic angle is accurately represented by a polynomial, the method provides a good description of the variation of the lengths of all directions within the crystal while ensuring complete internal consistency between all calculated cell dimensions at a given *P* and *T*.

### 2.6. Triclinic crystal system

In the triclinic system there are six cell parameters and the volume, of which six are required to define the geometry of the lattice and unit cell. Thus it appears that if EoSs are defined for *a*, *b* and *c* and also for the three *d* spacings 100, 010 and 001, then the unit cell is completely defined. However, this is not the case, because the relationships between the unit-cell angles and the *d* spacings take the form

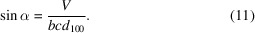




**Figure 8:**
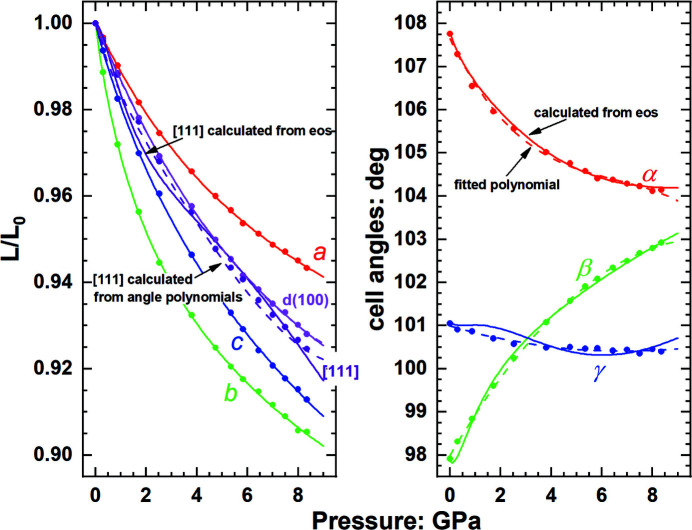
Unit-cell-parameter and angle variation of triclinic C_4_H_4_N_3_Cu_2_I_2_ with pressure (Conesa-Egea *et al.*, 2017 ▸). Solid lines for *a*, *b* and *c* are the fitted axial EoSs. The angles calculated (solid lines) from these axial EoSs and the EoSs for three *d* spacings and the volume do not match the measured data. As a consequence, the evolution of directions such as [111] is predicted to have unrealistic variations with pressure (solid line). The use of polynomial functions to describe the evolution of the angles with pressure (dashed lines) together with the same axial EoS for *a*, *b* and *c* reproduces (dashed lines) the measured variation of *d*(100) and [111] with pressure.

This defines the unit-cell angle α in terms of *V*, *d*_100_ and two unit-cell parameters, but the unit-cell angles are required to calculate the volume through equation (1). If one wants to retain the advantages of using only EoSs to describe the unit cell it is therefore necessary specify all seven EoSs for the three cell axes, three principal *d* spacings and volume. This introduces a spurious degree of freedom because the cell parameters and volume are treated independently and therefore the self-consistency between the EoSs is not ensured. This approach also suffers from the same problem of uncertainties in the unit-cell angles if they approach 90° (Fig. 8 ▸), which then leads to the prediction of unphysical behaviour of certain directions within the crystal, such as the [111] lattice vector shown in Fig. 8 ▸.

The alternative approach is to use a separate polynomial for each of the unit-cell angles, in which case the unit-cell is uniquely and consistently determined in combination with EoSs for three of the four parameters *a*, *b*, *c* and *V*. Fig. 8 ▸ shows that this provides a more accurate description of the variation of lengths of different lattice vectors and *d* spacings within the unit cell of triclinic crystals. The volume calculated from the six cell parameters is indistinguishable from the EoS fitted to the volume, at the scale of the measurement uncertainties in the data.

### 2.7. Implementation in *EosFit*

These procedures have been implemented in the EoS module of the *CrysFML* Fortran subroutine library (Rodriguez-Carvajal & Gonzalez-Platas, 2003 ▸), including the description of unit-cell angles by polynomials. Calculations can be performed with the CELL utility of the *EosFit7c* program. EoSs for volumes and cell parameters and, for triclinic crystals, the EoSs of the principal *d* spacings can be loaded from *EosFit* .eos files or be input directly by the user. Polynomials to describe the variation of the unit-cell angles in monoclinic and triclinic crystals can also be stored in, and loaded from, .eos files. The current version supports simple polynomials up to third order in *P* and *T*, thus allowing a maximum of ten coefficients of all orders up to *P* ^3^ and *T* ^3^, together with three cross-terms *PT*, *PT* ^2^ and *P* ^2^ *T*. The program architecture will allow extension to higher-order polynomials or the implementation of other functions to describe angle variation, if these are required.

Apart from the constraints imposed by the symmetry of the crystal system, for example that *a* = *b* in uniaxial systems when the unique axis is specified as *c*, the choice of which unit-cell parameter to calculate from the EoSs of the others is a matter of user choice. Therefore, the program only checks and restricts the choice of EoSs to those consistent with the crystal system. Once a set of EoSs have been loaded, the program checks also for internal consistency of the EoSs, for example that the pressure scales and the units for cell parameters and volume are consistent between all of the EoSs. Calculations are prevented until the EoSs necessary for the crystal system are loaded. The available commands include the calculation and output of the full unit-cell parameters at a *P* and *T* chosen by the user, and the output to a file listing the cell parameters over a range of *P* or *T*. The full properties (length, modulus, thermal expansivity *etc*.) of any individual direction can be calculated, as well as lines in *P*–*T* space of constant length (*i.e.* the linear equivalents of isochors of volume) and lines of constant ratio between a pair of directions. Pressure can be calculated from the length of any direction or *d* spacing in the crystal. The results of all of these calculations can be written into text files so that the calculated values can be imported into plotting software.

With the approach described here there are four different types of directions (or volume) whose properties are calculated in different ways. The properties of unit-cell axes (or the volume) for which EoSs are loaded, or which are symmetrically equivalent to a cell axis for which an EoS has been loaded, are calculated directly from their EoSs. All of the properties of other unit-cell axes or the volume (*i.e.* those without a loaded EoS) are calculated directly from the loaded EoSs from the other axes through the relationships given in equations (1)–(9). For other, general, directions in the unit cell the cell parameters are first calculated from the loaded EoSs and the metric tensor is constructed, from which the length of the chosen vector is calculated. Properties such as the linear modulus or thermal expansivity are calculated, numerically from a spline to a series of length calculations over a small range of *P* or *T*, as appropriate. This numerical approach, equivalent to that used by Paufler & Weber (1999 ▸) and Langreiter & Kahlenberg (2015 ▸), means that the calculated properties of general directions have lower precision than those calculated for unit-cell axes for which EoSs are available, and the precision decreases as the order of the volume derivative increases; calculated values of the linear modulus *M* are more reliable than the derivatives *M*′ or 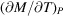 .

## 3. Tensor properties

The directions in a crystal structure of maximum and minimum thermal expansion or compression are given by the eigenvectors of the corresponding second-rank tensors. The eigenvectors are also called the principal axes of the tensor (Nye, 1957 ▸). In crystals of orthorhombic or higher symmetries the eigenvectors are constrained to be fixed in direction to be parallel to the axes of the conventional unit cell, and only their magnitude is a property of the crystal structure. The magnitudes of the principal axes, and the corresponding tensor components, are therefore given by the compressibility and thermal expansivity of the corresponding unit-cell axes, which are in turn defined by their axial EoSs.

In monoclinic crystals one principal axis is constrained to lie parallel to the diad axis. The other two principal axes lie in the plane perpendicular to the diad axis, but their directions in this plane are not constrained by symmetry and they can therefore rotate around the unique axis with a change in *P* or *T*. In triclinic crystals there are no constraints on the directions of the principal axes of these property tensors, meaning that they are completely free to rotate. In these cases, the full tensors must be calculated. The traditional method was to first calculate the strains between a pair of determinations of the unit cell of the crystal at different *P* or *T* (*e.g.* Ohashi & Burnham, 1973 ▸; Schlenker *et al.*, 1978 ▸) and then divide the strains by the *P* or *T* increment between the measurements to obtain the compressibility or thermal expansion tensors. This approach has several widely recognized disadvantages [discussed by Jessen & Küppers (1991 ▸), Paufler & Weber (1999 ▸) and Knight (2010 ▸)]. First, it returns a property tensor that is an average over the finite *P* or *T* range between the two data points. Second, the exact values of the tensor components are dependent on the initial and final conditions; thermal expansivity tensors calculated across different temperature intervals with the same mid-point temperature will have different values of their components. Third, in cases where there are significant changes in the unit-cell angles between the two unit cells, the directions of the eigenvectors of the strain and property tensors are defined relative to the reference cell under the assumption that the Cartesian axial system used to describe the tensors is fixed and does not rotate during the *P* and *T* change. As a consequence, the directions of the eigenvectors relative to the cell at the mid-point conditions are undefined, and a different choice of reference cell will lead to principal axes with slightly different directions relative to the underlying crystal structure.

To avoid these problems, Paufler & Weber (1999 ▸) developed the alternative approach of defining the thermal expansion tensor directly in terms of the derivatives of the unit-cell parameters with respect to temperature. They used polynomials to determine the unit-cell-parameter changes with temperature, from which the temperature derivatives are derived analytically and the thermal expansion tensor is unambiguously defined for any temperature within the range of the data. The values of the tensor components and the directions of the eigenvectors are not averages but are defined at the same temperature as one another (Paufler & Weber, 1999 ▸; Langreiter & Kahlenberg, 2015 ▸). The same approach can be applied to determine the compressibility tensor from a series of unit-cell determinations with pressure (Knight, 2010 ▸). The method does not rely on the algebraic form of the equations used to describe the cell-parameter variations, so the thermal expansion and compressibility tensors can be calculated from the temperature and pressure derivatives of the linearized EoSs of crystals.

### 3.1. Implementation in *EosFit*

In the *EoS* module of the *CrysFML* library (Rodriguez-Carvajal & Gonzalez-Platas, 2003 ▸) we implement the principles of the method of Paufler & Weber (1999 ▸). In crystal systems of higher than monoclinic symmetry, the only non-zero tensor components for the conventional choices of orientation of the Cartesian axes are the diagonal components. These are calculated directly from the set of self-consistent linearized EoSs fitted to the unit-cell parameters, with the components of the compressibility tensor being the inverse of the corresponding linear moduli. In monoclinic and triclinic crystal systems, the values of the tensor components also depend on the choice of orientation of the Cartesian axes relative to the crystallographic axes, and on the derivatives of real and reciprocal angles. If the real-space angles are described by polynomial functions, their derivatives are taken directly from the functions. Otherwise their derivatives are calculated by splines over a small range of *T* and *P* around the point at which the tensor is required. The derivatives of the reciprocal-space angles are always calculated by splines. The equations of Paufler & Weber (1999 ▸) provide the equations for one orientation of the Cartesian axes, those of Tribaudino *et al.* (2011 ▸) for a second, and those for two further commonly used orientations of the Cartesian axes were derived from these and coded into the *CrysFML* library. All three possible orientations of monoclinic unit cells are also supported.

The CELL utility of *EosFit7c* allows the user to calculate the property tensors from a set of self-consistent linearized EoSs. Facilities are provided to list the tensor components over a range of *P* or *T*, along with the eigenvalues of the properties and the directions of the principal axes (eigenvectors) relative to the Cartesian axes and the real and reciprocal unit-cell axes. A calculation of the nearest low-index plane normal and lattice vector is also provided to aid the user to relate the directions of the principal axes to the structure of the crystal. If the original unit-cell data are available, the results can be compared with the property tensors calculated by the finite difference between consecutive cell determinations by the STRAIN utility of *EoSFit7c*. Note that the STRAIN utility supports several different strain definitions (Zotov, 1990 ▸), which should become equivalent in the infinitesimal limit represented by the derivative approach used here.

## 4. Conclusions

We have proved algebraically in equations (1)–(9) that the volume variation of a crystal with *P* and *T* predicted from its unit-cell parameters described by conventional linearized (axial) EoSs is never exactly the same as that from a volume EoS, unless the crystal is cubic. Using independent EoSs for the symmetrically distinct unit-cell parameters and the volume is therefore always physically inconsistent and introduces false additional degrees of freedom. This applies to all types of EoS. The physical inconsistency arises from the anisotropy in the pressure derivatives of the axial compressibilities. Whether or not this is a significant issue for a given crystal depends on the degree of elastic anisotropy, the range of compression of interest, and the precision of experimental data or the precision and internal consistency required in calculations. Discrepancies are greatest for highly anisotropic soft materials such as many metal–organic frameworks and molecular crystals. If internal consistency is important, we have described how the full variation of the unit-cell parameters, volumes and elastic properties of a crystal can be described by suitable combinations of the axial and volume EoSs for each crystal system, and we have provided the tools for such calculations in the 2021 release of the *EosFit7c* program. The consistency of such calculations has been demonstrated with examples from all crystal systems. The example of zircon demonstrates that the method is also useful for determining the elastic properties of very stiff directions in a crystal that are too stiff to determine precisely by direct experimental measurement.

## Figures and Tables

**Table 1:** Properties at reference conditions of an example tetragonal crystal that obey the symmetry constraints on elastic properties

	*a* axis, *b* axis	*c* axis	Volume
*L*_0_, *V*_0_ (Å Å^3^)	5.0	4.0	100.0
*M*_0_, *K*_0_ (GPa)	40.0	20.0	10.0
M_0’, K_0’	5.0	20.0	5.625
